# Development of a Duplex Insulated Isothermal PCR Assay for Rapid On-Site Detection and Differentiation of Genotypes 1 and 2 of African Swine Fever Virus

**DOI:** 10.3389/fcimb.2022.948771

**Published:** 2022-07-07

**Authors:** Ruilong Song, Penggang Liu, Yang Yang, Hu Suk Lee, Changhai Chen, Xiaodong Wu, Xiangdong Li

**Affiliations:** ^1^ Jiangsu Co-innovation Center for Prevention and Control of Important Animal Infectious Diseases and Zoonoses, College of Veterinary Medicine, Yangzhou University, Yangzhou, China; ^2^ Joint International Research Laboratory of Agriculture and Agri-Product Safety, The Ministry of Education of China, Yangzhou University, Yangzhou, China; ^3^ International Livestock Research Institute (ILRI), Hanoi, Vietnam; ^4^ Jiangsu Provincial Center for Animal Disease Control and Prevention, Nanjing, China; ^5^ National African Swine Fever (ASF) Reference Laboratory, National Exotic Animal Disease Center, China Animal Health and Epidemiology Center, Qingdao, China

**Keywords:** onsite detection, African swine fever virus, genotype I and II, point-of-care, duplex insulated isothermal PCR

## Abstract

Genotype II African swine fever virus (ASFV) has been plaguing Asian pig industry since 2018. Recently, genotype I ASFV was reported for the first time in China. Since there is no commercial vaccine available against ASFV, early onsite detection and quick culling procedures are commonly used by many countries all over the world. It is important that the above two genotypes of ASFV could be quickly differentiated during onsite detection at the same time. In this study, we established a sensitive and simple Fluorescent Probe Hydrolysis-Insulated isothermal PCR (iiPCR) that can detect and differentiate two genotypes of ASFV within 40 minutes. The positive or negative results of tested samples were displayed on the screen of the device automatically after PCR amplification was complete. The detection limit of the iiPCR was tested to be 20 copies for both genotype I and genotype II ASFVs. There was no cross-reactivity with other swine viruses by using the established iiPCR. Fifty-eight ASFV positive samples confirmed by National ASF Reference Laboratory were subjected to the established duplex iiPCR for genotype differentiation. The results showed that all these ASFV-positive samples belong to genotype II. At last, we found serum samples could be directly used as the templates for iiPCR without comprising sensitivity and specificity. Therefore, the duplex iiPCR established in study provide a useful tool for ASFV onsite detection and genotype differentiation.

## Introduction

African swine fever virus (ASFV), the sole member of the family *Asfarviridae*, is the causative agent of African swine fever (ASF) and can infect domestic pigs, wild boars and warthogs with typical clinical signs of high fever, lethargy, digestive dysfunctions, and abortion with high mortality (Li and Tian, 2018). The first case of ASF was reported in Kenya in the 1920s and since then it has been prevalent in Africa, Europe, and Asia (Mushagalusa et al., 2021). China has reported the first ASF case in 2018, and hundreds of cases in different provinces were officially confirmed which led to huge economic losses to Chinese swine industry (Zhou et al., 2018).

ASFVs could be divided into 24 genotypes based on the viral *B646L* gene. The first reported ASFV strain in China belongs to genotype II (Zhao et al., 2019). In the past three years, genotype II ASFV with different patterns of gene deletions were frequently reported, which lead to the emerging field strains with different virulence (Gao et al., 2021). Most recently, genotype I ASFV was reported in China and the infected pigs had milder clinical symptoms with lower mortality (Sun et al., 2021a). PCR and real-time PCR are commonly used techniques for ASFV diagnosis. However, most of these developed PCRs were designed based on the conserved *p72* gene and therefore could not differentiate different genotypes of ASFV (Pikalo et al., 2022). We previously developed a duplex real-time PCR assay based on *E296R* gene which could simultaneously detect genotype I and genotype II ASFVs (Li et al., 2022).

Insulated isothermal PCR (iiPCR) is recently used to detect several veterinary viruses (Lung et al., 2016; Zhang et al., 2019). Spontaneous fluid convection is triggered in a capillary tube and the denaturation, annealing, and extension of PCR are completed with high efficiency in iiPCR reaction (Tsai et al., 2012). The assay has the equivalent sensitivity as real-time PCR and displays positive or negative results on the device screen after data processing. Zhou et al. recently developed a universal ASFV iiPCR based on *p72* gene (Zou et al., 2022). It only takes 25 min to get the results with similar sensitivity to that of real-time PCR, but is a simpler and more rapid method for ASFV field surveillance. In this study, we aim to develop a duplex iiPCR that can detect and differentiate genotype I and genotype II ASFVs simultaneously.

## Methods

### Primers and Probes

The primers and probes of the duplex iiPCR were based on *E296R* gene of ASFVs as previously described (Li et al., 2022). The sequences of primers and probes were listed in **Table 1**. The two TaqMan probes were labeled with VIC and FAM at the 5′ ends, respectively. The primers and probe were synthesized from Sangon (Shanghai, China).

**Table 1:** Primers and probes of the duplex iiPCR in this study (Li et al., 2022).

Primers	Sequence	Channel
Genotype I-Primer-Forward	ATTAGTTTTACACCTAGGCGCC	
Genotype I-Probe	*VIC*-GCCTGAACTTATCGTGGA-MGB	550 nM
Genotype I-Primer-Reverse	CTGCTCCATTGTACTGTATTTATATG	
Genotype II-Primer-Forward	GTTAGTTTTACACCTAGGCGCT	
Genotype II-Probe	*FAM*-GCCTGAACTTATTATGGA-MGB	520 nM
Genotype II-Primer-Reverse	CTGCTCAATTGTACTGTATTTATATG	

### Development and Optimization of the Duplex iiPCR

Commercial Uni-ii PCR Starter kit (GeneReach Biotechnology) was used to develop the duplex iiPCR. Various concentrations of components in iiPCR reaction were tested and optimized in a total volume of 50 μL. Optimization of the established iiPCR was performed and determined by the absorption ratios at 520 nM and 550 nM according to the manufacture’s instruction.

**Figure 1:**
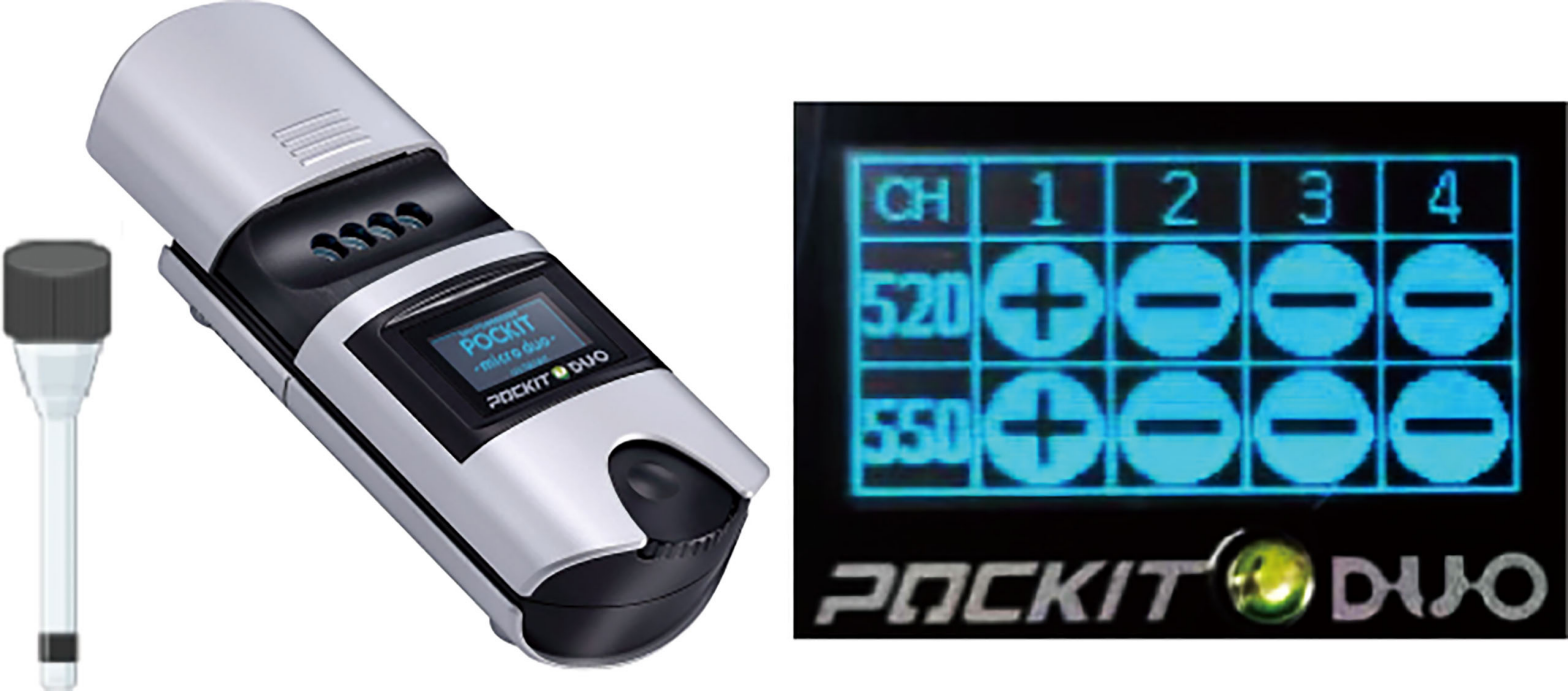
Illustration of the duplex iiPCR. The iiPCR tube was put into POCKIT™ Micro Duo device and the default program was performed. The results showed on the screen with “+” for positive sample or “-” for negative sample automatically.

The iiPCR was carried out within POCKIT™ Micro Duo device (GeneReach Biotechnology) and the default program of 42°C for 10 min and 95°C for 30 min was conducted. Fluorescent signals were collected, and signal-to-noise (S/N) ratios were calculated by dividing signals collected after iiPCR by those from before iiPCR. The results were finally displayed as “+” or “-” for positive and negative samples (**Figure 1**).

### Sensitivity and Specificity of the Duplex iiPCR

The PCR products of two pairs of primers were cloned into pUC57 vector working as the positive plasmid. The concentration was converted into copy numbers using the following formula: y (copies/μL) = (6.02 × 10^23^) × (x(ng/μL) × 10^−9^ DNA)/(DNA length × 660). The sensitivity of the duplex ii PCR was tested by using ten-fold diluted standard positive plasmid from 1-1000 copies/μL as templates in this study as previously described (Li et al., 2022).

The specificity of the duplex iiPCR was determined by testing DNA samples of ASFV genotype I (the nucleic acid imported from Spain by China Animal Health and Epidemiology Center in 2015) and genotype II (preserved in National ASF Reference Laboratory). DNA/cDNA of other porcine viruses including classical swine fever virus (CSFV), pseudorabies virus (PRV), porcine reproductive and respiratory syndrome virus (PRRSV), porcine circovirus 2 (PCV2), porcine parvovirus (PPV), porcine epidemic diarrhea virus (PEDV) was preserved in our laboratory. DNA samples were extracted from different tissues of clinical healthy pigs using the Ambion^®^MagMAXTM Total Nucleic Acid Isolation Kit (Applied Biosystems).

### Application of the Duplex iiPCR on Clinical Samples

Fifty-eight ASFV positive DNA samples extracted from clinical samples including lymph node (5), liver (2), lung (3), spleen (7), serum (25), and environmental swab (16) were provided by NARL (**Table 4**) and were used to differentiate ASFV genotype I and II using the established duplex iiPCR in this study. These ASFV positive DNA samples were previously verified by the real-time PCR recommended by NARL. The ASFV genotype I DNA samples extracted from E70, Ba71V, and E75 strains were imported from Spain by China Animal Health and Epidemiology Center in 2015. Twenty-five ASFV positive serum samples (same samples within 58 clinical samples mentioned above) were preserved in NARL. Another 10 serum samples were collected from healthy pigs as negative controls. These serum samples were inactivated and tested directly as the templates for iiPCR without DNA extraction. Two microliters of these serum samples were used in the iiPCR reactions. The study was performed in strict accordance with the regulations in the guide for the care and use of laboratory animals of the China Animal Health and Epidemiology Center and Yangzhou University.

## Results

### Optimization of the Duplex iiPCR

ASFV positive and negative DNA samples were used to set up the iiPCR using commercial Uni-ii PCR Starter kit as the manufacture’s instruction. Various concentrations of primers and probes were tested and optimized with Taq DNA polymerase and 2×Uni-ii buffer provided by the kit with a final reaction volume of 50 μL. The S/N ratio of non-template blank with ASFV positive DNA samples reached maximum when iiPCR reaction components consisted of forward and reverse primers at 10 μMol (2 μL for each primer), 10 μMol probe (1 μL for each probe), 5 U/μL Taq DNA polymerase (2 μL), 2×Uni-ii buffer (25 μL), DNA template (2 μL), and dH_2_O (11 μL). The above parameters were used in the following experiments.

### Sensitivity and Specificity of the Duplex iiPCR

The plasmid DNA was 10-time serially diluted into 1~1000 copy numbers/μL and were used to test the sensitivity of iiPCR. As shown in **Table 2**, the limits of detection for genotype I and genotype II were both at 20 copy numbers/reaction, which was consistent with the results of NARL-recommend real-time PCR. The results suggested the high sensitivity of the established duplex iiPCR. To assess the specificity of iiPCR, nucleic acids extracted from other swine viruses including CSFV, PRV, PRRSV, PCV2, PPV, and PRoV were uses as templates. RNA of these RNA viruses was reverse transcribed into cDNA before performed iiPCR. Also, nucleic acids extracted from different tissues of healthy pigs including lymph nodes, liver, lung, spleen, and sera were used. As shown in **Table 3**, positive signals were only generated from ASFV positive DNAs but not from DNAs of other swine virus or healthy pigs, which indicated good specificity of the established duplex iiPCR.

**Table 2:** Sensitivity of the duplex iiPCR. .

Copy number/reaction	Genotype I (550 nM)			Genotype II (520 nM)			NARL-recommend Real-time PCR (Ct)		
2000	+	+	+	+	+	+	+ (31.12)	+ (30.55)	+ (31.8)
200	+	+	+	+	+	+	+ (34.77)	+ (34.25	+ (34.51)
20	+	+	+	+	+	+	+ (37.16)	+ (37.58)	+ (36.99)
2	–	–	–	–	–	–	- (N/A)	- N/A)	- (N/A)

Each dilution of plasmids was tested for 3 times. The cut-off Ct value for NARL-recommend real-time PCR was set as 38 according to the manual instruction.

**Table 3:** Specificity of the duplex iiPCR.

DNA/cDNA of viruses	Duplex iiPCR	
	Genotype I550 nM	Genotype II520 nM
ASFV Genotype I	+	–
ASFV Genotype II	–	+
CSFV	–	–
PRV	–	–
PRRSV	–	–
PCV2	–	–
PPV	–	–
PRov	–	–
Lymph node DNA of healthy pig	–	–
Liver DNA of healthy pig	–	–
Lung DNA of healthy pig	–	–
Spleen DNA of healthy pig	–	–
Serum DNA of healthy pig	–	–

### Application of the Duplex iiPCR on ASFV Positive DNA Samples

Fifty-eight clinical samples including 2 lymph node samples, 2 liver samples, 3 lung samples, 7 spleen samples, 25 serum samples, and 16 environmental swab samples, which has previously been sent to NARL in 2021, were confirmed to be ASFV positive by NARL-recommend real-time PCR. To further differentiate ASFV genotype I and II of these samples, the established duplex iiPCR in this study was used to detect DNA extracted from these clinical samples. As shown in **Table 4**, all these DNA samples were confirmed to be ASFV genotype II, which was consistent with the results of a duplex ASFV real-time PCR reported previously (Li et al., 2022). DNA samples extracted from E70, Ba71V, and E75 strains were tested to be ASFV genotype I as expected.

**Table 4:** Application of the duplex iiPCR on clinical ASFV positive DNA samples.

Sample No.	DNA extracts from positive samples	NARL-recommend real-time PCR (Ct value)	Duplex iiPCR	
			Genotype I (550nM)	Genotype II (520nM)
1	Lymph node	32.20	–	+
2	Lymph node	28.54	–	+
3	Lymph node	33.01	–	+
4	Lymph node	18.66	–	+
5	Lymph node	19.84	–	+
6	Liver	19.66	–	+
7	Liver	17.13	–	+
8	Lung	18.36	–	+
9	Lung	22.14	–	+
10	Lung	15.45	–	+
11	Spleen	23.13	–	+
12	Spleen	21.93	–	+
13	Spleen	22.47	–	+
14	Spleen	16.89	–	+
15	Spleen	16.32	–	+
16	Spleen	14.95	–	+
17	Spleen	16.92	–	+
18	Serum	22.31	–	+
19	Serum	15.71	–	+
20	Serum	20.14	–	+
21	Serum	22.47	–	+
22	Serum	17.26	–	+
23	Serum	18.55	–	+
24	Serum	35.98	–	+
25	Serum	24.29	–	+
26	Serum	25.44	–	+
27	Serum	25.67	–	+
28	Serum	18.05	–	+
29	Serum	16.31	–	+
30	Serum	15.77	–	+
31	Serum	19.38	–	+
32	Serum	17.68	–	+
33	Serum	18.17	–	+
34	Serum	23.42	–	+
35	Serum	19.15	–	+
36	Serum	20.54	–	+
37	Serum	22.85	–	+
38	Serum	25.90	–	+
39	Serum	13.79	–	+
40	Serum	32.66	–	+
41	Serum	17.84	–	+
42	Serum	30.79	–	+
43	Environmental swab	33.12	–	+
44	Environmental swab	26.89	–	+
45	Environmental swab	26.24	–	+
46	Environmental swab	31.38	–	+
47	Environmental swab	29.74	–	+
48	Environmental swab	32.69	–	+
49	Environmental swab	35.74	–	+
50	Environmental swab	33.55	–	+
51	Environmental swab	36.42	–	+
52	Environmental swab	36.65	–	+
53	Environmental swab	33.10	–	+
54	Environmental swab	32.67	–	+
55	Environmental swab	34.89	–	+
56	Environmental swab	33.92	–	+
57	Environmental swab	25.94	–	+
58	Environmental swab	24.01	–	+
/	E70 DNA	18.56	+	–
/	Ba71V DNA	21.80	+	–
/	E75 DNA	24.91	+	–
/	Pos Ctrl	25.44	+	+
/	Neg Ctrl	N/A	–	–

### Application of the Duplex iiPCR Directly on Serum Samples

Zhou, et al. reported blood samples could be directly used as the template for iiPCR without compromising the sensitivity of assay (Zou et al., 2022). Therefore, the 25 ASFV positive serum samples in the 58 clinical samples and 10 ASFV negative serum samples were inactivated and used to test if the serum samples could be directly used in our established duplex iiPCR. As shown in **Table 5**, all these ASFV positive serum samples (No.1-25) showed positive results which was consistent with the results of their corresponding DNA samples. All these ASFV negative serum samples were tested to be negative (No.26-35). The above results suggested serum samples could be directly used as iiPCR templates without DNA extraction.

**Table 5:** A Direct application of serum samples without DNA extraction and tested on the duplex iiPCR.

Sample No.	Duplex iiPCR	
	Genotype I (550nM)	Genotype II (520nM)
1	–	+
2	–	+
3	–	+
4	–	+
5	–	+
6	–	+
7	–	+
8	–	+
9	–	+
10	–	+
11	–	+
12	–	+
13	–	+
14	–	+
15	–	+
16	–	+
17	–	+
18	–	+
19	–	+
20	–	+
21	–	+
22	–	+
23	–	+
24	–	+
25	–	+
26	–	–
27	–	–
28	–	–
29	–	–
30	–	–
31	–	–
32	–	–
33	–	–
34	–	–
35	–	–
Pos Ctrl	+	+
Neg Ctrl	–	–

To test sensitivity of the duplex iiPCR using serum samples as templates, three genotype II ASFV positive serum samples with different real-time PCR Ct values in **Table 4** (No.42 Ct=30.79, No.3 Ct=33.01, and No.24 Ct=35.98) were selected and tested. These three serum samples were 10-fold serially diluted with ASFV negative serum working as the templates for iiPCR. In parallel, the nucleic acids were extracted from 200 µl of the serum samples and eluted with same volume of TE buffer. As shown in **Table 6**, the detection limits for both unextracted or extracted samples were the same, which indicated the unextracted serum samples could be directly used as templates in the established duplex iiPCR. Unfortunately, we could not perform the above test on genotype I ASFV since we do not have any positive serum samples.

**Table 6:** Sensitivity comparison of the duplex iiPCR using unextracted or extracted ASFV genotype II positive serum sample.

Dilution	Unextracted ASFV positive serum			Extracted DNA		
	No.42	No.3	No.24	No.42	No.3	No.24
Undiluted	+/-	+/-	+/-	+/-	+/-	+/-
10^-1^	+/-	+/-	-/-	+/-	+/-	-/-
10^-2^	+/-	-/-	-/-	+/-	-/-	-/-
10^-3^	-/-	-/-	-/-	-/-	-/-	-/-

Three serum samples with different Ct values detected by real-time PCR in Table4 were used. The Ct value of each sample was shown under the number of serum samples. The results of each sample were shown by reading at 520/550 nM on the device.

## Discussion

ASFV has been prevalent in China in the past several years and led to huge economic losses to Chinese pig industry. Georgia-07-like genotype II ASFV has been the predominant strain in China since 2018 (Sun et al., 2021b). Recently, genotype I ASFV emerged in Shandong and Henan provinces with lower pathogenicity but high transmissibility (Sun et al., 2021a). To date, there is no commercial vaccine available to prevent and control this disease. The most effective measure to control ASF is early diagnosis combined with stamping-out policy (Galindo and Alonso, 2017).

Currently, different types of real-time PCR were developed widely used for ASFV diagnosis (Pikalo et al., 2022). *P72, CD2v*, and *MGF360* genes are commonly targeted in those developed real-time PCR assays which could differentiate ASFV wild strains and gene-deleted strains (Guo et al., 2020). However, these methods could not differentiate ASFV genotype I and II. Our research group recently developed a duplex real-time PCR based on *E296R* gene which could simultaneously detect and differentiate genotype I and genotype II ASFV strains (Li et al., 2022). However, the real-time PCR can only be performed by specialized personals in the laboratory settings and could not be applied to onsite point-of-care testing. Since early diagnosis of ASFV is the first and most crucial step, a rapid, sensitive and field-deployable method is a desirable tool for ASFV control.

iiPCR relies on the fluorescent probe hydrolysis driven by natural liquid convection in a capillary tube with the equivalent sensitivity and specificity as real-time PCR. However, the denaturation, annealing, and extension of PCR are automatically completed within a capillary tube with high amplification efficiency within a short period of time. An iiPCR based on ASFV *p72* gene was recently developed, which only take 25 minutes to read with equivalent sensitivity as real-time PCR (Zou et al., 2022). Therefore, in this study, we established a duplex iiPCR generating two fluorescence signals (VIC and FAM) that can detect and differentiate genotype I and II ASFVs simultaneously.

POCKIT™ Micro Duo device was used in this study due to its two fluorescence channels. The developed duplex iiPCR showed high specificity and sensitivity, which indicated the assay can provide accurate and producible detection. ASFV positive DNA samples stored at National ASF Reference Laboratory was used to test the duplex iiPCR. All these samples were tested to be genotype II, which indicates genotype II ASFV is still the predominant genotype in China.

DNA or RNA samples need to be extracted from clinical samples before applying as the templates for loop-mediated isothermal amplification (LAMP) and recombinase polymerase amplification (RPA), the procedures that hander the onsite application of these methods in the field (Lung et al., 2016; Zhang et al., 2019). It will be desirable if the body fluid samples such as sera could be directly used as PCR templates without nucleotide acid extraction. Serum DNA could be released when the capillary tube is heated within a thermally baffled iiPCR device with top temperature of 95°C. Therefore, we used serum samples directly as the PCR templates in the established duplex iiPCR. Consistent with the results of extracted DNA samples, all ASFV positive serum samples were tested to be positive without compromising the sensitivity, which solved the bottleneck problem of nucleotide acid extraction for iiPCR application in the field. Of note, we found the hemoglobin in the serum samples may lead to the false positive results in the established iiPCR. Therefore, the hemolytic blood samples could not be directly used as temples.

Due to the pre-set program of 42°C for 10 min and 95°C for 30 min on POCKIT™ Micro Duo device, which includes reverse transcript and amplification procedures, the total time for this duplex iiPCR is 40 min. If the reverse transcript cycle program could be removed, it will only take 30 min to read with comparable sensitivity as real-time PCR did. More importantly, POCKIT™ Micro Duo displays positive or negative results on the device screen, and users can immediately get the final results without specific training. Therefore, the established duplex iiPCR in this study will be a useful tool for onsite detection and differentiation of the two genotypes of ASFV.

## Data Availability Statement

The original contributions presented in the study are included in the article/supplementary material. Further inquiries can be directed to the corresponding authors.

## Author Contributions

XL and XW conceived the experiments. RS, PL, YY, and CC performed the experiments. XL, HL, and XW analyzed the data. XL wrote the manuscript. All authors contributed to the drafting of the manuscript and proof reading of the submitted version. All authors contributed to the article and approved the submitted version.

## Funding

This work was supported by Jiangsu Agricultural Science and Technology Independent Innovation Fund Project [CX(21)2035], Jiangsu Provincial Key R&D plan (BE2020398), Jiangsu Agricultural Industry Technology System (JATS [2021] 358), and the Project of the Priority Academic Program Development of Jiangsu Higher Education Institutions (PAPD).

## Conflict of Interest

The authors declare that the research was conducted in the absence of any commercial or financial relationships that could be construed as a potential conflict of interest.

## Publisher’s Note

All claims expressed in this article are solely those of the authors and do not necessarily represent those of their affiliated organizations, or those of the publisher, the editors and the reviewers. Any product that may be evaluated in this article, or claim that may be made by its manufacturer, is not guaranteed or endorsed by the publisher.
